# Enoxaparin preserves cellular morphology and modulates mitophagy-associated Parkin–LC3 in acute ischemic rat myocardium

**DOI:** 10.1590/1806-9282.20251136

**Published:** 2025-12-15

**Authors:** Fevzi Ayyıldız, Uğur Karagöz, Tünay Kurtoğlu, Pınar Akokay, Çağatay Bilen

**Affiliations:** 1 Aydın Adnan Menderes University, Faculty of Medicine, Department of Cardiovascular Surgery – Aydın, Türkiye.; 2 İzmir Kavram Vocational School, Department of Medical Laboratory Techniques – İzmir, Türkiye.

**Keywords:** Enoxaparin, Myocardial ischemia, Cell size, Mitophagy

## Abstract

**OBJECTIVE:**

Despite the longstanding clinical use of low-molecular-weight heparins and their well-characterized mechanisms of action, their effects on mitophagy pathways in acute myocardial ischemia remain incompletely delineated. The aim of this study was to investigate the enoxaparin effect on cardiomyocyte morphology and the immunopositivity of mitophagy-associated Parkin–LC3 proteins in a rat model of acute myocardial ischemia.

**METHODS:**

Female Wistar albino rats (median weight: 400 g, minimum: 375 g-maximum: 415 g) were divided into sham (n=7), control (n=7), and treatment (n=7) groups. The control group received subcutaneous 0.9% saline (0.2 mL/kg) twice daily for 28 days, followed by the induction of myocardial ischemia. The treatment group was administered subcutaneous enoxaparin sodium (1 mg/kg) twice daily for the same duration before undergoing myocardial ischemia. Myocardial ischemia was induced by occluding the proximal left anterior descending coronary artery using a 6-0 polypropylene suture for 10 min. Morphometric calculations of cardiomyocytes (cell length and diameter) were performed using light microscopy. Immunohistochemical analysis was performed using anti-Parkin and anti-LC3 antibodies, and immunopositive cells were counted.

**RESULTS:**

Statistically significant differences were found between the groups (p<0.001). Post-hoc analysis revealed significant differences between the control and treatment groups based on cell length, cell diameter, Parkin, and LC3 values (p<0.001, p=0.001, p<0.001, and p=0.001, respectively).

**CONCLUSION:**

This study demonstrated that enoxaparin preserved cardiomyocyte morphology and reduced the number of Parkin–LC3 immunopositive cells in a model of induced acute ischemic injury in rat myocardium.

## INTRODUCTION

In acute ischemic myocardium, cardiomyocytes exhibit oncotic necrosis morphology^1^. The current medical treatments for myocardial ischemia focus on limiting the necrotic area. Mitophagy and subsequent mitochondrial regeneration processes play a crucial role in preserving cellular integrity during myocardial ischemia.

The impairment of mitochondrial membrane potential (ΔΨm) leads to the interaction of PTEN-induced putative kinase 1, a mitochondrial surface protein, with the ubiquitin ligase Parkin protein, initiating the mitophagy process^2^. Ubiquitin-tagged mitochondria bind to microtubule-associated protein 1 light chain 3 (LC3), facilitating autophagosome formation. Lysosomes then fuse with mitochondrial autophagosomes to degrade the damaged mitochondria.

Parkin expression, a key mitophagy mediator protein, increases during ischemia^3^. Parkin-mediated mitophagy reduces oxidative stress and limits myocardial necrosis^4^. Activation of Parkin is suggested to be an adaptive mechanism that mitigates cardiomyocyte death induced by myocardial ischemia and hypoxia^3^.

Low-molecular-weight heparins (LMWHs), particularly enoxaparin, are recommended for reducing the incidence of ischemic events in patients with acute coronary syndromes^5^. Anticoagulant agents are also essential components of preoperative medical management for patients scheduled for coronary revascularization who are at high risk of ischemia.

Despite their longstanding clinical use and well-characterized mechanisms of action, the effects of LMWHs on mitophagy-associated proteins in acute myocardial ischemia remain incompletely understood. This study examined the effects of enoxaparin on cell morphology and Parkin–LC3 immunopositivity in the ischemic rat myocardium.

## METHODS

This experimental study using a myocardial ischemia model was approved by the Local Ethics Committee for Animal Experiments at Aydın Adnan Menderes University (date: April 25, 2024; approval no. 64583101/2024/48). All procedures were carried out in accordance with the European Communities Council Directive of November 24, 1986 (86/609/EEC). The rat model of myocardial ischemia was selected because of its ready availability, reliability, reproducibility, and the impracticality of performing similar interventions in humans. The suitability of this model for studying mitophagy and autophagy has been well-established in the literature^1,6^. Female Wistar albino rats (median weight: 400 g, minimum: 375 g-maximum: 415 g) were obtained from the Experimental Animals Breeding and Research Laboratory of the Faculty of Medicine at XXXXX University and housed in the same facility. All experiments were performed under standard laboratory conditions (12 h light/12 h dark cycle; temperature 21°C±1°C; humidity 55%±5%). Animals had ad libitum access to tap water and pelleted feed and were housed in standard polycarbonate cages with daily welfare monitoring. The criteria for exclusion included ≥15% loss of body weight, impaired food and water intake, unresponsiveness to stimuli, and signs of bleeding. With 95% power and a 5% error margin, an effect size of d=0.94 was achieved for a three-group study with seven observations per group. After numbering, rats were randomly allocated (simple randomization) into three groups: sham (Group 1, n=7), control (Group 2, n=7), and treatment (Group 3, n=7). Group 1 did not undergo any intervention or ischemia. Group 2 received subcutaneous injections of 0.9% saline (0.2 mL/kg) twice daily for 28 days and underwent myocardial ischemia. Group 3 received subcutaneous enoxaparin sodium (1 mg/kg; Oksapar-KoçakFarma, Istanbul, Turkey) twice daily for the same duration before the induction of myocardial ischemia.

### Surgical procedures and induction of myocardial ischemia

Surgical procedures were performed on all animals (n=21) under anesthesia induced by intraperitoneal injection of xylazine hydrochloride (10 mg/kg; Rompun-Bayer, Istanbul, Turkey) and ketamine hydrochloride (50 mg/kg; Ketalar-Pfizer, Istanbul, Turkey). Adequate anesthesia was confirmed by the loss of corneal and pedal withdrawal reflexes. The animals were placed in the supine position on the operating table, and oxygen was administered via a face mask at a flow rate of 2 L/min. The surgical field was prepared by hair removal and povidone–iodine antisepsis. A surgical loupe (3.5× magnification; Xenosys, Incheon, Korea) was used throughout the surgery. After a midline skin incision and median sternotomy, cardiac exposure was achieved. In Groups 2 and 3, the proximal left anterior descending artery was occluded with a 6/0 polypropylene suture for 10 min to induce ischemia. Regional dyskinesia, tissue edema, and discoloration were evaluated as indicators of ischemia. Euthanasia by exsanguination was performed immediately after cardiac exposure in Group 1 and following the ischemia procedure in Groups 2 and 3. Hearts were harvested, rinsed with 0.9% saline, and fixed in 10% neutral-buffered formalin for subsequent histopathological and immunohistochemical analyses.

### Light microscopy protocol

Hearts were fixed in 10% buffered formalin for 48–72 h. Following fixation, tissues were dehydrated using a graded series of ethanol, cleared with xylene, and embedded in paraffin. Serial sections of 5 μm thickness were prepared using a rotary microtome (Leica RM-2255; Nussloch, Germany) and mounted on poly-L-lysine-coated slides. The sections were deparaffinized, rehydrated, and stained with hematoxylin and eosin, Masson's trichrome, and Picro-Sirius Red^6-8^. Histological images were analyzed using a computer-assisted image analysis system consisting of a microscope (Olympus BX-51; Tokyo, Japan) equipped with a high-resolution digital camera (Olympus DP-71; Tokyo, Japan). Morphometric measurements were performed using ImageJ software. Measurements were performed on 10 cells from 5 different areas for each subject. Cardiomyocyte length and diameter were measured, and groups were compared based on these parameters.

### Determination and evaluation of Parkin and LC3

Immunohistochemical analysis was performed using the streptavidin–biotin method^7^. Tissue sections were mounted on poly-L-lysine-coated slides and incubated overnight at 60°C. After deparaffinization with a xylene series and rehydration with graded ethanol, antigen retrieval was performed in 10 mM citrate buffer at 95°C for 5 min. Sections were circumscribed with a Dako pen (Dako Aps; Glostrup, Denmark) and treated with 3% hydrogen peroxide at 37°C for 15 min to block endogenous peroxidase activity. Nonspecific binding was prevented by incubation with a normal serum-blocking solution for 30 min.

Next, sections were incubated overnight in a humidified chamber (30–60% humidity) with primary antibodies against LC3 (BiossUSA, LC3 Polyclonal Antibody, Cat. No: BS-4843R) and Parkin (BiossUSA, Parkin Polyclonal Antibody, Cat. No: BS-1865R). The following day, after washing with phosphate-buffered saline, the sections were sequentially incubated with biotinylated IgG and streptavidin–peroxidase conjugate (SensiTek; West Logan, USA, HRP Anti-Polyvalent Lab Pack, Cat. No: SHP125). Immunoreactivity was visualized using 3,3′-diaminobenzidine (Roche Diagnostics; Basel, Switzerland, Cat. No: 11718096001) for 2 min. Finally, slides were counterstained with Mayer's hematoxylin (Sigma-Aldrich; Ohio, USA) for 10 s and mounted using Entellan (Merck; Darmstadt, Germany). For the quantitative assessment of Parkin and LC3, 100 cells were randomly selected and counted from different regions in each group. The percentage of cells with positive Parkin and LC3 immunoreactivity was then calculated. To ensure objective evaluation, all assessments were performed by a histologist who was blinded to the study protocol.

### Statistical analysis

All calculations and evaluations were performed using IBM SPSS Statistics 26 (IBM Corp., Released 2019). The normality of distribution was assessed using the Shapiro-Wilk test. Notably, three-group comparisons were conducted by one-way analysis of variance for normally distributed data. The homogeneity of variances was tested using Levene's test; when homogeneity was violated, Welch's test was applied. For significant overall differences, pairwise comparisons were performed using Tukey's test when the variances were homogeneous and Tamhane's T2 test otherwise. Quantitative data were presented as mean±standard deviation and median (minimum-maximum). A p-value of <0.05 was considered statistically significant.

## RESULTS

**Figure 1:**
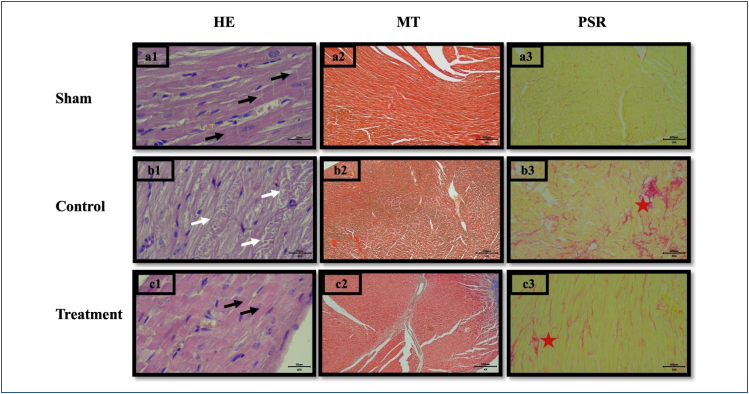
Histological sections of sham (a1, a2, and a3), control (b1, b2, and b3), and treatment groups (c1, c2, and c3). Hematoxylin and eosin (HE) staining: 40×, bar 50 μm (a1, b1, and c1). Masson trichrome staining: 4×, bar 500 μm (a2, b2, and c2). Picro-Sirius Red staining: 10×, bar 200 μm (a3, b3, and c3).

**Figure 2:**
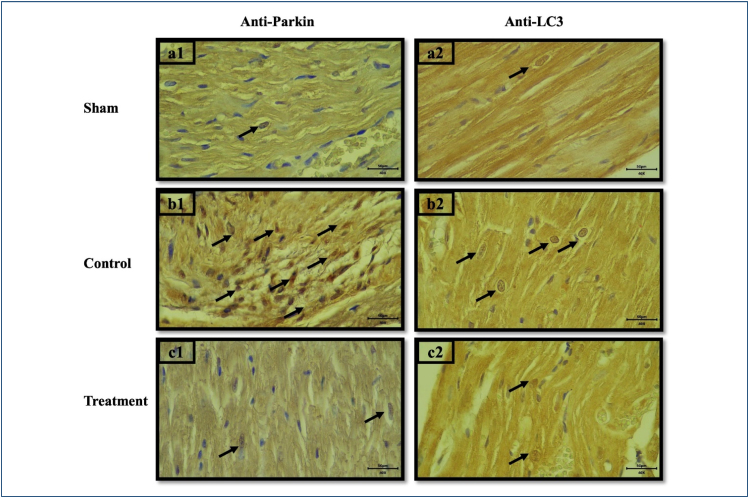
Histological sections of Parkin and LC3 staining. a1—sham group, 40×, b1—control group, 40×, and c1—treatment group, 40×. Parkin staining: a2—sham group, 40×, b2—control group, 40×, and c2 treatment group—40×, LC3 staining. Parkin- and LC3-positive cells marked with black arrow.

Throughout the study period, all 21 rats completed the protocol without any losses. The light microscopy images are presented in Figure 1. In the sham group, cardiomyocytes were regularly aligned, nuclei were centrally located, and intercalated disks (black arrows) were prominent. In the control group, vacuolization (white arrows) and disarray were evident, with poorly defined cell borders. In the treatment group, cell borders were distinct and nuclei were relatively well centered, resembling the sham group. Masson's Trichrome and Picro-Sirius Red staining revealed normal histomorphological fibrillar architecture with regular undulation in the sham and treatment groups, whereas the control group exhibited increased collagen fibrils (red asterisks). Immunopositive cells for Parkin and LC3 are presented in Figure 2.

There were statistically significant differences among the groups in cell length, cell diameter, and Parkin and LC3 immunopositivity (p<0.001 for all) (Table 1). Post-hoc analysis revealed significant differences between the control and treatment groups in terms of cell length, cell diameter, Parkin, and LC3 values (p<0.001, p=0.001, p<0.001, and p=0.001, respectively) (Table 2).

**Table 1:** Comparison of variables between groups.

	Control (n=7)	Sham (n=7)	Treatment (n=7)	Test stat.	p-value
Cell length (μm)	87.82±7.64	63.34±4.55	70.37±4.05	34.982	**<0.001** f
Cell diameter (μm)	16.31±1.57	11.3±0.81	13.51±1.11	30.478	**<0.001** f
Parkin (+ cells n/100)	56.14±12.58	8.71±2.36	18.57±8.56	47.519	**<0.001** w
LC3 (+ cells n/100)	55.86±13.99	10.86±1.35	19.86±6.89	38.069	**<0.001** w

f One-way ANOVA test;

w Welch's test;

Mean±SD. Bold values indicate statistically significant differences (p<0.05).

**Table 2:** Pairwise comparison results.

	Control vs. sham (p-value)	Control vs. treatment (p-value)	Sham vs. treatment (p-value)
Cell length‡	**<0.001**	**<0.001**	0.077
Cell diameter‡	**<0.001**	**0.001**	**0.008**
Parkin†	**<0.001**	**<0.001**	0.065
LC3†	**<0.001**	**0.001**	**0.039**

‡ Tukey's post-hoc test;

† Tamhane's T2 post-hoc test.

Bold values indicate statistically significant differences (p<0.05).

## DISCUSSION

The results of this study demonstrated that enoxaparin preserved cardiomyocyte morphology and reduced Parkin–LC3 immunopositivity in acute ischemic myocardial injury in rats. These findings suggest that enoxaparin may exert cellular cardioprotective effects against myocardial ischemia and modulate mitophagic activity in the ischemic myocardium.

Accumulating evidence indicates that beyond anticoagulant effects, LMWHs exhibit pleiotropic actions through the suppression of inflammation and reactive oxygen species (ROS) generation, preservation of nitric oxide, and maintenance of vascular homeostasis^9-11^. Although anticoagulant mechanisms are activated rapidly, non-anticoagulant effects may require prolonged exposure to fully manifest^12^.

Following the onset of an ischemic attack, cardiomyocytes experience rapid oxygen depletion, thereby compromising mitochondrial ATP production. Contractile function is reduced and anaerobic metabolism, which has limited compensatory capacity, takes over. Sustained ATP deficiency triggers two critical sequelae: (1) Na^+^/K^+^-ATPase pump inactivation, leading to intracellular Na^+^-H_2_O accumulation and cellular swelling. (2) Loss of sarcolemmal ion gradients and mitochondrial membrane potential^1^.

Cell swelling is followed by necroptosis, resulting in cell necrosis^1^. In this study, the significant increase in cardiomyocyte length and diameter observed in the control group was consistent with oncotic necrosis on morphological examination. These findings parallel previous reports indicating that early ischemic remodeling is characterized by increased cellular thickness and disproportional changes in cardiomyocyte morphology^13^. In contrast, the preservation of cellular morphology in the enoxaparin-treated group indicates the attenuation of early hypoxic injury.

During ischemia, incomplete reduction of oxygen within cells accelerates ROS formation and exacerbates mitochondrial damage^14^. After acute ischemic injury, cells may follow one of two pathways: cell death or survival via mitophagy^1^. Although alternative pathways become more prominent in the ischemic myocardium, the core mitophagy pathway—dependent on ubiquitin (Parkin–LC3)—is activated^2,15^. Parkin-mediated mitophagy may function as an adaptive response that limits hypoxia-associated cellular injury in myocardium^3^.

We observed a reduced Parkin and LC3 immunopositivity in enoxaparin-treated animals. However, we cannot discriminate mitochondrial Parkin translocation, and the LC3 antibody collectively recognizes both LC3-I and LC3-II isoforms. Autophagosome formation is specifically correlated with elevated LC3-II, so our findings cannot provide direct evidence of mitophagic flux^16^. Nevertheless, these results suggest that enoxaparin may limit hypoxia-related damage in cardiomyocytes and reduce the need for mitophagy.

Future therapeutic strategies for myocardial ischemia are likely to focus on mitophagy regulation, as in many other diseases^17^. In this regard, elucidating the effects of enoxaparin and similar anticoagulant agents on mitophagic processes in myocardial ischemia is an important area for further research. In this context, if confirmed in future studies, enoxaparin may also be considered as a factor that reduces ischemic injury in the myocardium, alongside exercise, whose cardioprotective effects are well- established^18^.

### Limitations

This study has several limitations. First, antioxidant and anti-inflammatory mechanisms that may mediate the potential cardioprotective effect of enoxaparin against ischemia were not assessed. Additionally, to more precisely elucidate mitophagic activity, advanced analyses targeting Parkin behavior and LC3 subtypes—such as electron microscopy and Western blotting—are required. Finally, it should be noted that because the data obtained are based on an experimental model, the results should be interpreted with caution when extrapolating the findings to clinical settings.

## CONCLUSION

The results obtained in the present study demonstrate that enoxaparin treatment preserves cardiomyocyte morphology and reduces Parkin- and LC3-immunopositive cell counts in an acute ischemic injury model of the rat myocardium. These findings suggest that, in addition to its established cardioprotective effects against ischemia, enoxaparin may also modulate the mitophagic response. Further mechanistic studies are required to validate these findings.

## Data Availability

The datasets generated and/or analyzed during the current study are available from the corresponding author upon reasonable request.
